# Percutaneous Transfistulous Interventions for Intractable Pancreatic Fistula

**DOI:** 10.1155/2011/109259

**Published:** 2011-03-30

**Authors:** Masahiko Hirota

**Affiliations:** Department of Surgery, Kumamoto Regional Medical Center, 5-16-10 Honjo, Kumamoto 860-0811, Japan

## Abstract

Three techniques for the treatment of intractable pancreatic fistula: percutaneous transfistulous pancreatic duct drainage (PTPD), percutaneous transfistulous pancreatojejunostomy (PTPJ), and percutaneous transfistulous pancreatic duct embolization (PTPE) are presented as treatment options for intractable pancreatic fistula. PTPD is effective for most cases of intractable fistula that communicate with the main pancreatic duct. However, PTPD itself is not enough in some specific cases. PTPJ and PTPE are applicable in such cases.

## 1. Introduction

Pancreatic fistula (PF) remains a significant problem in the management of pancreatectomy. Despite decreased morbidity and mortality rates after pancreatic resection, PF remains a common and potentially lethal complication after pancreatectomy. Its reported incidence varies from 6 to 38% [1–7]. If PF occurs, the pancreatic juice must be drained externally to avoid the development of intra-abdominal hemorrhage and sepsis. Eighty five to 95% of PF can be managed by a combination of external drainage and medical therapy [3, 8]. However, occasional patients with inadequate treatment may require further advanced treatments.

PF also sometimes becomes a significant problem in severe acute pancreatitis. Pseudocysts and postnecrotic collections of the pancreas are treated by percutaneous drainage if they are large and likely to aggravate the patients' condition. Approximately 65–90% of patients recover from this disease by percutaneous drainage alone [9–12]. However, recovery is often difficult if the main pancreatic duct is injured [9, 10]. 

Techniques of percutaneous transfistulous drainage of the main pancreatic duct (percutaneous transfistulous pancreatic duct drainage: PTPD) for intractable PF cases are presented. Furthermore, percutaneous transfistulous pancreatojejunostomy (PTPJ) and percutaneous transfistulous pancreatic duct embolization (PTPE) are also presented as advanced techniques of PTPD.

## 2. Techniques

### 2.1. Percutaneous Transfistulous Pancreatic Duct Drainage (PTPD)

**Figure 1:**
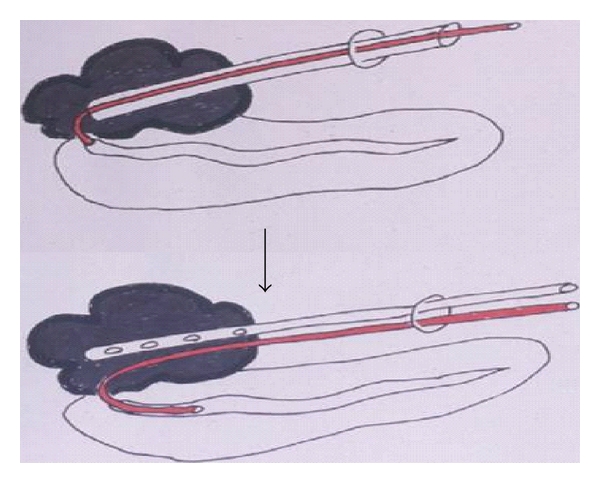
Percutaneous transfistulous pancreatic duct drainage (PTPD). Using the abscess drainage tube as a sheath, an angled catheter is inserted into the main pancreatic duct.

This technique is useful for major PF in which the main pancreatic duct is visualized in fistulography [13, 14]. Any abscess due to PF is first drained percutaneously. An angled angiography catheter (RIM or RC2, Medikit, Tokyo) is inserted into the main pancreatic duct via the injured site (Figure 1). By twisting and taking the catheter in and out of the fistula, the route into the pancreatic duct can be selected. The abscess drainage tube can be used as a sheath of the catheter. After placing the catheter into the main pancreatic duct, the abscess content is drained using another catheter. Because almost all pancreatic juice drained externally with this system, the abscess is cured within a short period.

### 2.2. Percutaneous Transfistulous Pancreatojejunostomy (PTPJ)

**Figure 2:**
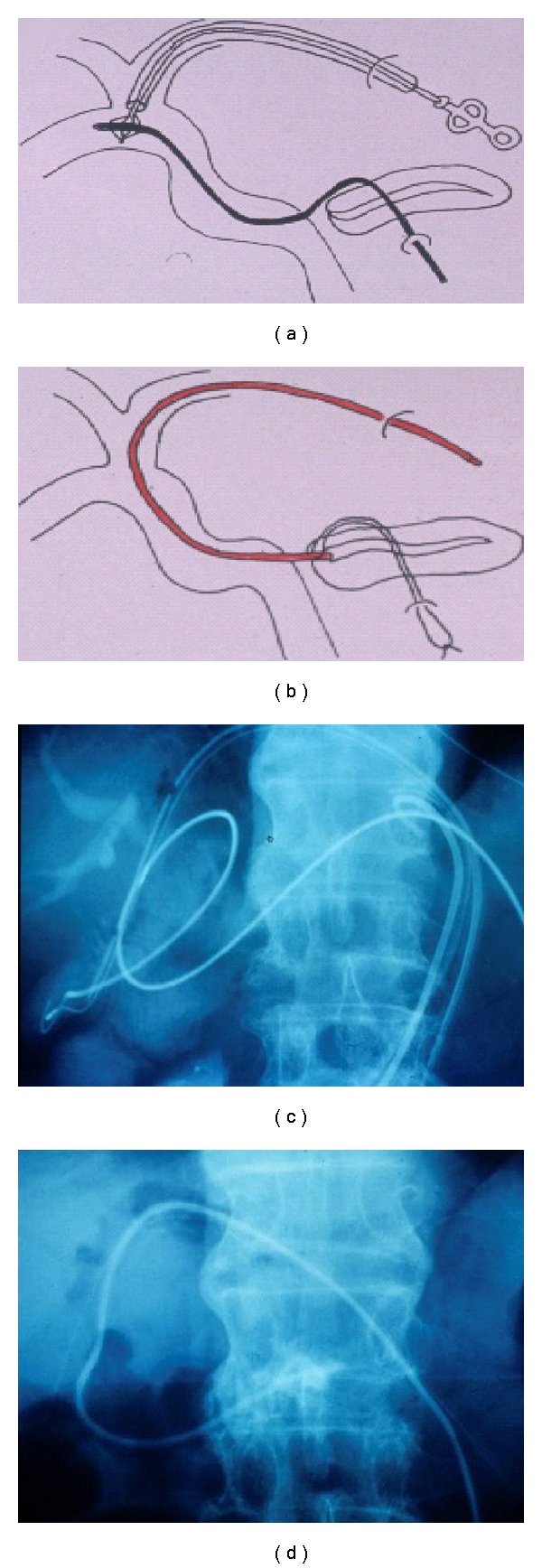
Percutaneous transfistulous pancreatojejunostomy (PTPJ). Firstly, abscess around the pancreatojejunostomy is drained percutaneously. Then, drainage catheters are inserted into the pancreatic duct (PTPD procedure) and jejunum, respectively. A guide wire inserted into the jejunum via the drainage tube is grasped by a basket catheter and led externally from percutaneous transhepatic biliary drainage (PTBD) route (a and b). A drainage catheter is inversely inserted into the remnant pancreatic duct over the guide wire (c and d).

When intractable PF is developed after pancreatoduodenectomy, abscess around the pancreatojejunostomy is drained percutaneously at first. Then, drainage catheters are inserted into the pancreatic duct (PTPD procedure) and jejunum, respectively. A guidewire is inserted into the jejunum via percutaneous transfistulous route through the drainage tube. The guidewire, grasped by a basket catheter inserted from the percutaneous transhepatic biliary drainage (PTBD) route under fluoroscopy, is led externally via the PTBD outlet (Figures 2(a) and 2(b)). Finally, a drainage catheter is inversely inserted into the remnant pancreatic duct over the guidewire via the PTBD route and the jejunum (Figures 2(c) and 2(d)). The position of the drainage catheter is adjusted by pulling a thread placed on its tip through abscess drainage outlet. Pancreatic juice then flows along the drainage catheter from the pancreas to outside of the body via the jejunum and intrahepatic bile duct. After one month, the drainage catheter is removed, and PTPJ is established [13].

### 2.3. Percutaneous Transfistulous Pancreatic Duct Embolization (PTPE)

**Figure 3:**
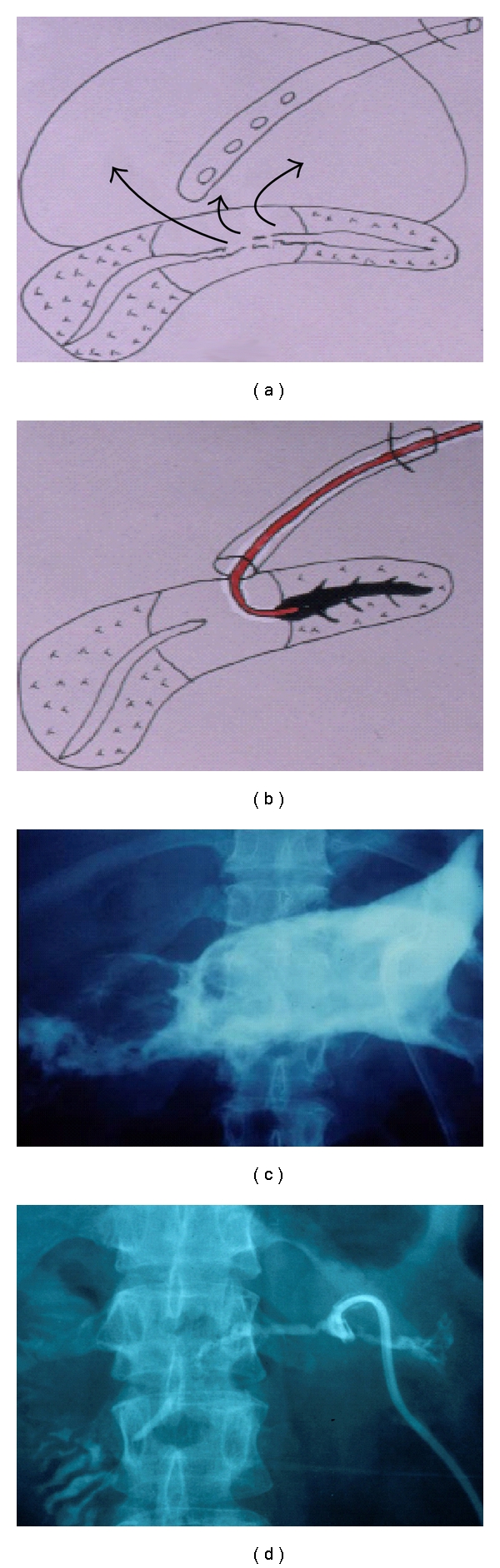
Percutaneous transfistulous pancreatic duct embolization (PTPE). Large postnecrotic collection is formed owing to disruption of the main pancreatic duct at the body (a and b). The upstream pancreatic duct is inserted with a drainage catheter (PTPD procedure) (c and d) and is injected with prolamine via the catheter under microscopy.

Large postnecrotic collection can be formed owing to disruption of the main pancreatic duct in some severe necrotizing acute pancreatitis (Figures 3(a) and 3(b)). In such cases, PF may be intractable. We radically treat such PF by selective drainage of the upstream pancreatic duct via the damaged site of the pancreatic duct (PTPD procedure) (Figures 3(c) and 3(d)). The upstream pancreatic duct is embolized with prolamine (Ethibloc, Ethicon GmbH, Norderstedt, Germany) under fluoroscopy to extinguish the exocrine function of the caudal pancreas (PTPE procedure). Prolamine is a gel that hardens within minutes when it contacts with water. It has been used for the embolization of the pancreatic duct to prevent the development of PF after resection or transplantation of the pancreas [15, 16]. The PF can be cured by several PTPE procedures [14].

## 3. Discussion

PF represents clinically relevant complications with life-threatening consequences (i.e., bleeding, sepsis) after pancreatic resection and in acute necrotizing pancreatitis. PTPD, PTPJ, and PTPE are presented as treatment options for intractable PF. Visualization of the main pancreatic duct during fistulography indicates that the PF is intractable.

The conventional treatment strategy for PF consists of establishing adequate external drainage, treating infection, providing nutritional support, and reducing pancreatic juice secretion by parenteral nutrition or octreotide. With effective drainage, most of the PF can be cured by conservative treatment. However, the management of intractable PF cases has not been standardized and remains challenging. The management of intractable PF is individualized. Several procedures have been reported, including endoscopic drainage, percutaneous intervention [13], and surgical treatment such as completion pancreatectomy. Occlusion of the pancreatic duct has also been performed using tissue glues, such as prolamine, ethylene-vinyl alcohol, and fibrin glue [14, 17]. The vast majority of PFs, which are characterized by maintained continuity with an unobstructed main pancreatic duct (side fistula) respond well to conservative treatment [18]. However, PFs that lose continuity with the downstream (end fistula) do not respond to conservative treatment. 

Transpapillary stents can bypass the high resistance of the sphincter of Oddi, ductal strictures, and calculi, thereby reducing the intraductal pressure that is the driving force behind the fistula. Endoscopic pancreatic sphincterotomy is also recommended for the treatment of PF after DP [17]. However, the communication routes between the duodenum and pancreatic duct that are created by these procedures may sometimes exaggerate infection. From this standpoint, percutaneous transfistulous drainage (PTPD procedure), if possible, is preferable. 

In necrotizing pancreatitis, disruption of main pancreatic duct appears to be associated with the most severe form of necrosis [19, 20]. Uomo et al. [21] reported that the incidence of main pancreatic duct injury is 31%. If the main pancreatic duct is injured in necrotizing pancreatitis, intractable PF may be developed. Such conditions are often difficult to treat. However, drainage of the distal pancreatic duct using an angiographic catheter under fluoroscopy (PTPD procedure) followed by extinction of the exocrine function of the distal pancreas by PTPE is highly effective in treating intractable PF even in such cases. Such condition may be the most appropriate indication of PTPE.
